# *Rickettsia felis* in *Aedes albopictus* Mosquitoes, Libreville, Gabon

**DOI:** 10.3201/eid1810.120178

**Published:** 2012-10

**Authors:** Cristina Socolovschi, Frédéric Pagés, Didier Raoult

**Affiliations:** Aix-Marseille Université, Marseille, France (C. Socolovschi, D. Raoult);; and CIRE/ARS Océan Indien (Les Cellules interrégionales d’épidémiologie/Agence Régionale de Santé), La Réunion, France (F. Pagés)

**Keywords:** Rickettsia felis, rickettsioses, transitional group rickettsiae, Aedes albopictus, Asian tiger mosquito, mosquito, Gabon, vector, bacteria, vector-borne infections

**To the Editor:** *Rickettsia felis*, an emerging pathogen first identified in the cat flea (*1*), has been detected in other fleas, ticks, mites, and booklice (*2*). *R. felis* can be cultured in mosquito cell lines derived from *Anopheles gambiae* and *Aedes albopictus* (Asian tiger) mosquitoes (*2*), so its compatibility with mosquitoes in nature can be suspected. In sub-Saharan Africa, *R. felis* bacteremia in humans is common¸ especially during the rainy season, when mosquitoes proliferate. We tested anthropophilic mosquitoes for the presence of *R. felis* DNA (*3*–*5*).

During December 2008–January 2010, we randomly selected female *Ae. albopictus* and *Ae. aegypti* mosquitoes (96 each) from specimens obtained by human-landing collections from 4 sites in Libreville, Gabon (*6*). Specimens were collected during the rainy season (mid-January–end of May and end of September–mid-December); no parity data were available.

We extracted 192 DNA samples from homogenate (abdomen, wings, legs) of each nonengorged, host-seeking, adult mosquito by using the BioRobot 8000 (QIAGEN S.A.S., Courtaboeuf, France) and QIAamp Media MDx Kit (QIAGEN) according to the manufacturer’s instructions. Samples were screened by quantitative real-time PCR (qPCR) targeting the biotin synthase (*bio*B) gene (*4*). Positive results were confirmed by qPCR-based molecular detection targeting the *orf*B gene, which codes for a transposition helper protein. This qPCR used a set of primers not previously used in our laboratory (R_fel.OrfB_F: 5′-CCCTTTTCGTAACGCTTTGCT-3′ and R_fel.OrfB_R: 5′-GGGCTAAACCAGGGAAACCT-3′) and the probe R_fel.OrfB_P: 6-FAM-TGTTCCGGTTTTAACGGCAGATACCCA-TAMRA. Specificity of the qPCR was tested in silico and on the 31 *Rickettsia* spp. from our laboratory. The final qPCR reaction mixture contained extracted DNA (5 μL) and mix (15 μL) that contained master mix (10 μL) from the QuantiTect Probe PCR Kit (QIAGEN, Hilden, Germany), each primer (0.5 μL, 20 pmol), probe (0.5 μL, 62.5 nmol), and RNase-free water (3.5 μL). Amplification and sequence detection were performed in a CFX96 Touch thermocycler (Bio-Rad, Marnes-la-Coquette, France) as follows:15 min at 95°C followed by 40 cycles of 1 s at 95°C, 40 s at 60°C, and 40 s at 45°C.

Test results for all *Ae. aegypti* homogenates were negative for *R. felis* DNA. Of the 96 *Ae. albopictus* specimens, 3 (3.1%) had positive test results for the *R. felis* species–specific real-time qPCR and the confirmatory qPCR, with mean cycle thresholds ± SDs of 37.34 ± 1.7 (*bio*B gene; mean copies/mosquito 5 × 10^2^ [minimum 1.2 × 10^2^, maximum 1.4 × 10^3^]) and 33.64 ± 1.4 (*orf*B gene; mean copies/mosquito 5 × 10^2^ [minimum 1.5 × 10^2^, maximum 1 × 10^3^). One of the 3 samples was collected in January and 2 in March. The samples came from 3 different districts of Libreville (Akebe Poteau, Alibandeng, Camp des Boys) and were tested by nested PCR targeting the citrate synthase (*glt*A) gene (*7*). *Rickettsia montanensis* DNA was used as a positive control. Sequencing was performed as described (*7*), and ChromasPro version 1.34 (Technelysium Pty Ltd., Tewantin, Queensland, Australia) was used to analyze sequence data. Sequences of the *bio*B (120/120) and *glt*A (1,230/1,230) amplicons at the nucleotide level were 100% homologous to sequences for *R. felis* URRWXCal2 (GenBank accession no. CP000053). The *glt*A fragment sequence was deposited in GenBank (accession no. JQ674484). Mosquitoes were considered positive for *R. felis* when the qPCR result was <35 cycle thresholds for 1 target gene and the additional DNA sequence was successfully amplified. No sample in this study was positive for only 1 target gene or had a qPCR threshold >35 cycle thresholds for both genes.

Contamination is a critical problem for the PCR-based identification of microbes. However, the validity of the data we report is based on strict laboratory procedures and controls that are commonly used in the World Health Organization Reference Center for Rickettsial Diseases, including rigorous positive and negative controls to validate the test. Each positive qPCR result was confirmed by another *R. felis*–specific qPCR (*orf*B) not previously used in our laboratory (to avoid contamination with other amplicons).

*Ae. albopictus* mosquitos are native to Southeast Asia, colonizing rural and peri-urban sites. In Gabon, *Ae. albopictus* was the vector for outbreaks of chikungunya and dengue virus infections (*6*). Our study indicates that mosquitoes can carry *R. felis*, and the prevalence and load (1.8% –70% and 1.3 × 10^3^–1.6 × 10^7^, respectively) detected in mosquitoes in this study are consistent with the low-end range of those detected in cat fleas, the confirmed biological vector and reservoir (*8*,*9*).

We investigated the presence of *Rickettsia* spp. in mosquitoes neglected as possible vectors of rickettsial diseases (*2*). Other *Aedes* spp. and other genera of mosquitoes should be tested. The role of mosquitoes as *Rickettsia* spp. vectors remains to be demonstrated in additional studies that use the Mitchell criteria. These studies should include the use of cell culture to isolate or detect *R. felis* in salivary glands of specimens from wild-caught mosquitoes, PCR, immunofluorescence, and the fluorescence in situ hybridization technique; demonstration of infection of a mosquito after experimental feeding on a bacteremic host or bacterial suspension; and demonstration of the transmission of bacteria to a vertebrate through the bite of a mosquito (*10*).
